# MiR-185 Targets the DNA Methyltransferases 1 and Regulates Global DNA Methylation in human glioma

**DOI:** 10.1186/1476-4598-10-124

**Published:** 2011-09-30

**Authors:** Zuping Zhang, Hailin Tang, Zeyou Wang, Baoxin Zhang, Wei Liu, Hongmei Lu, Lan Xiao, Xiaoping Liu, Rong Wang, Xiaoling Li, Minghua Wu, Guiyuan Li

**Affiliations:** 1 Cancer Research Institute, Key Laboratory of Carcinogenesis and Cancer Invasion, Ministry of Education Central South University, 110 Xiang-Ya Road, Changsha 410078, Hunan, P.R. China; 2 Department of Parasitology, Central South University, Changsha 410078, Hunan, PR China; 3 Cancer Research Institute, University of South China, 28 Changsheng West Road, Hengyang 421001, Hunan, P.R. China; 4 Armed Police Hospital of Hunan Province, Changsha 410008, Hunan, P.R. China; 5 Research Center for Modernization of Chinese Herbal Medicine, College of Chemistry and Chemical Engineering, Central South University, Changsha 410083, PR China

**Keywords:** DNA methylation, MiR-185, Glioma, DNMT1

## Abstract

**Background:**

Perturbation of DNA methylation is frequent in cancers and has emerged as an important mechanism involved in tumorigenesis. To determine how DNA methylation is modified in the genome of primary glioma, we used Methyl-DNA immunoprecipitation (MeDIP) and Nimblegen CpG promoter microarrays to identify differentially DNA methylation sequences between primary glioma and normal brain tissue samples.

**Methods:**

MeDIP-chip technology was used to investigate the whole-genome differential methylation patterns in glioma and normal brain tissues. Subsequently, the promoter methylation status of eight candidate genes was validated in 40 glioma samples and 4 cell lines by Sequenom's MassARRAY system. Then, the epigenetically regulated expression of these genes and the potential mechanisms were examined by chromatin immunoprecipitation and quantitative real-time PCR.

**Results:**

A total of 524 hypermethylated and 104 hypomethylated regions were identified in glioma. Among them, 216 hypermethylated and 60 hypomethylated regions were mapped to the promoters of known genes related to a variety of important cellular processes. Eight promoter-hypermethylated genes (ANKDD1A, GAD1, HIST1H3E, PCDHA8, PCDHA13, PHOX2B, SIX3, and SST) were confirmed in primary glioma and cell lines. Aberrant promoter methylation and changed histone modifications were associated with their reduced expression in glioma. In addition, we found loss of heterozygosity (LOH) at the miR-185 locus located in the 22q11.2 in glioma and induction of miR-185 over-expression reduced global DNA methylation and induced the expression of the promoter-hypermethylated genes in glioma cells by directly targeting the DNA methyltransferases 1.

**Conclusion:**

These comprehensive data may provide new insights into the epigenetic pathogenesis of human gliomas.

## Background

Aberrant DNA methylation is associated with the genesis and progression of tumors [1]. Low levels of cytosine methylation in the genome (genomic hypomethylation) are accompanied by local locus-specific hypermethylation in cancer cells [2,3]. Genomic hypomethylation can lead to genome instability and proto-oncogene formation, which leads to their high expression [4]. On the other hand, the local promoter hypermethylation is usually associated with the functional silencing of tumor associated genes [5]. Therefore, cancer cells undergo massive alterations in their DNA methylation that results in abnormal gene expression and malignant phenotypes.

Glioma is the most frequent and devastating primary brain tumor in adults. Aberrant DNA methylation is associated with the development and progression of glioma [6]. The promoter hypermethylation and epigenetic silencing of the O6-methylguanine-DNA methyltransferase (MGMT) gene have been often described in glioma [7-9]. The promoter hypermethylation-associated silencing of other genes involved in the cell cycle [10], tumor suppression [11-15], DNA repair [16,17], tumor invasion [18], and apoptosis [19] have also been detected in malignant glioma. However, the aberrant genomic DNA methylation in glioma is still not fully understood.

DNA methyltransferases (DNMTs) are critical regulators of the status and intensity of methylation in the genome. Currently, three catalytically active DNMTs, namely DNMT1, DNMT3A, and DNMT3B, have been identified [20]. DNMT1 is the key maintenance methyltransferase in mammals and is responsible for both de novo and maintenance methylation of tumor-associated genes. DNMT3A and DNMT3B are predominately responsible for the de novo methylation. Although the mechanisms leading to aberrant DNA methylation remain to be fully elucidated, increased expression of DNMT1 and DNMT3B and decreased expression of DNMT3A have been observed in glioblastoma [21,22], suggesting that abnormal DNMT expression may contribute to aberrant DNA methylation and gliomagenesis.

MicroRNAs (miRNA) are ~20-22 nucleotide non-coding RNA molecules and can negatively regulate the gene expression. MicroRNAs can bind to the 3' untranslated region (3'UTR) of the target mRNA, trigger mRNA degradation and/or inhibit gene translation [23,24]. Growing evidence supports that miRNAs can act as both targets and effectors in the development of aberrant DNA methylation in the genome [25,26]. Silencing miRNA in the hypermethylation promoter of the genes occurs in various cancers [27-29]. Meanwhile, miRNAs can regulate DNA methylation by targeting the regulators of DNA methylation machinery [30,31]. The microRNA-185 is predicted to bind to the 3' UTR of DNA methyltransferases 1 (DNMT1). However, whether and how miR-185 could regulate DNMT1 expression and affect the genomic DNA methylation, contributing to the development of human glioma, has not been systemically explored.

The methlyation DNA immunoprecipitation-based chip analysis (MeDIP-chip) is a novel high-throughput array-based method using a monoclonal antibody against 5-methycytidine for the enrichment of the methylated DNA fragments and then hybridizing to the promoter and CpG islands of the entire human genome [32]. In this study, we employed the MeDIP-chip technology to investigate the methlyation status of whole-genome in glioma and normal brain tissues. Subsequently, the promoter methylation status of eight candidate genes was validated and the epigenetically regulated expression of these genes and the potential mechanisms were examined in human glioma samples. Our data indicate that low levels of miR-185 expression are associated the aberrant activation of DNMT1 and global DNA hypermethylation, contributing to the development of human glioma. More importantly, our findings indicate that miR-185 can directly target DNMT1, thereby leading to a reduction in global DNA methylation (GDM) and regulating the expression of the promoter-hypermethylated genes in glioma cells.

## Materials and methods

### Tissue samples

A total of 49 primary brain glioma samples were obtained from brain tumor patients randomly selected from the inpatient service of Xiangya Hospital, Central South University from 2006 to 2009. Individual patients with glioma were diagnosed, according to the criteria of World Health Organization (WHO), and their tumors were graded by pathologists based on the revised classification of the WHO (2007) [33]. The samples were snap-frozen immediately after resection and stored in liquid nitrogen until processing. Informed consent was obtained directly from individual patients and subject's relatives, and the experimental protocols were reviewed and approved by the Institute Research Board of the hospital. Forty glioma samples were composited of astrocytoma (grade I, grade II, grade III), glioblastoma multiform (grade IV) and oligoastrocytoma (grade II, grade III) and were used for methylation analysis by MassARRAY. Eleven brain white matter tissue samples from non-tumor patients were also collected as controls (six non-tumor brain samples and five epileptic brain samples). Of them, six primary glioma samples (astrocytoma grade I (n = 1), astrocytoma grade II (n = 3), and astrocytoma grade III (n = 2)) from three male and female patients and four non-tumor brain samples from two male and female were studied by MeDIP-chip.

### Cell lines

Human glioblastoma-derived cell lines, U251, U87, SF126 and SF767, were obtained from the Cell Research Institute of Peking Union Medical College (Peking, China). U251 and U87 were maintained in DMEM supplemented with 10% fetal calf serum (FCS) and standard antibiotics; SF126 and SF767 were cultured in minimal essential medium. All cells were cultured in a 37°C humidified incubator supplied with 5% CO_2_.

To study the effect of epigenetic modulation, glioma cell lines were treated with, or without, 5 μmol/L of 5-aza-2'-deoxycytidine (Sigma, Aldrich) for 4 days and the cells exposed to freshly prepared medium containing the same concentration of drug daily.

### MeDIP and microarray hybridization

The methylation status of global DNA of individual samples was determined by MeDIP-chip using the MeDIP-chip kit, according to the Nimblegen MeDIP-chip protocol. Briefly, genomic DNA was extracted from individual tissue samples and sonicated into 100-500 bp of random fragments. After heat-denaturation, individual DNA samples were probed with 5 μg of mouse anti-5-methylcytidine monoclonal antibody (Eurogenetec, San Diego, CA, USA) in IP buffer (0.5% NP40, 1.1% Triton X-100, 1.5 mM EDTA, 50 mM Tris-HCl, and 150 mM NaCl) with gentle rotation at 4°C overnight. Subsequently, the mixture of DNA and anti-5-methylcytidine was reacted with sheep anti-mouse IgG-conjugated magnetic beads (Bangs laboratories) at 4°C for 2 h. After washing, the beads were re-suspended and the bound proteins were digested with 80 μg of proteinase-K in digestion buffer (50 mM Tris, pH 8.0, 10 mM EDTA, 0.5% SDS) at 50°C for 3 h. The remaining DNA was extracted with phenol-chloroform and precipitated with ethanol. The precipitated DNA was re-suspended in 20 μl of 10 mM Tris-HCl pH 8.0 and used in quantitative real-time PCR (qRT-PCR) for the validation of IP efficiency and for microarray hybridization. The immunoprecipitated methylated DNA was labeled with Cy5 fluorophore and the input genomic DNA was labeled with Cy3 fluorophore. The labeled DNA samples were combined and hybridized to HG18 CpG promoter microarray 385 K (Nimblegen). After washing, the arrays were scanned using a GenePix 4000B scanner (Nimblegen). Data were extracted and exported to excel using GenePix Pro6.0.

### Microarray data analysis

Raw excel data files obtained from tiling array experiments were analyzed using NimbleScan™2.3 software. The ratios of Cy5 to Cy3 signals were calculated for all high-quality hybridization dots, normalized, and transformed to Log_2_Ratio. One-side Kolmogorov-Smirnov (KS) test was conducted to obtain *p value *and P value (-Log10 *p value*) of each probe, according to Log_2_Ratio of ambient probes within 750 bp sliding window width. Peak score was generated by interval analysis with a cutoff value of 2, maximum gap at 500 bp, and a minimum run with at least two consecutive probes. The regions with peak scores were defined as methylated, and the level of methylation was a positive correlation with peak scores. If the methylation frequency of a region in glioma was significantly higher than that in non-tumor brain tissues, we defined the region as a hypermethylation region. On the contrary, we defined it as a hypomethylation region. GFF files of Log_2_Ratio, P value, Peak score, and HG18 CpG Promoter Annotation data were exported to Signalmap 1.0 software for visual analysis and review.

### MassARRAY measurements of DNA methylation

The Sequenom MassARRAY platform (CapitalBio, Beijing, China) was used for the quantitative analysis of methylation. Briefly, genomic DNA was isolated from cell lines and brain tissue samples obtained from individual glioma patients and non-tumor subjects. The target DNA regions were amplified by PCR using bisulfite-modified DNA and specific primers. The PCR reactions were carried out in a total volume of 5 μL containing 1 pmol of each primer, 40 μM of dNTP, 0.1 unit of Hot Star Taq DNA polymerase (Qiagen), 1.5 mM MgCl_2_, and buffer. PCR amplifications were performed at 94°C for 15 min and subjected to 45 cycles of 94°C for 20 seconds, 62°C for 30 seconds, and 72°C for 1 min, followed by 72°C for 3 min. The remaining unincorporated dNTPs were dephosphorylated by shrimp alkaline phosphatase (SAP; Sequenom, San Diego, CA) at 37°C for 20 min and heat inactivated. The PCR products were directly used as template in a 7 μL transcription reaction. Twenty units of T7 DNA polymerase (Epicentre, Madison, WI) were used to incorporate either dCTP or dTTP in the transcripts. Ribonucleotides were used at 1 mM and the dNTP substrate at 2.5 mM; other components in the reaction were as recommended by the supplier. In the same step, RNase A (Sequenom) was added to cleave the *in vitro *transcripts. The mixture was then further diluted with H_2_O to a volume of 27 μL. Conditioning of the phosphate backbone prior to matrix-assisted laser desorption/ionization time-of-flight mass spectrometry (MALDI-TOF MS) was achieved by the addition of 6 mg CLEAN Resin (Sequenom). Further experimental analysis of the contents of DNA methylation was determined, as described previously [34].

### Chromatin immunoprecipitation

The potential protein and DNA interactions were characterized by chromatin immunoprecipitation using an EZ ChIP™ chromatin immunoprecipitation kit, according to the manufacturers' instruction (Upstate, USA). Briefly, U251, SF767, and SF126 glioma cells were cross-linked with 1% formaldehyde for 10 min and centrifuged. After being washed with ice-cold PBS, the cell pellets were lyzed in 1% SDS lysis buffer and sonicated. The cell lysates were incubated with 5 μg of control IgG antibody, anti-K4 trimethylated histone H3 antibody, anti-K9 trimethylated histone H3 antibody, or anti-acetylated histone H3 antibody (Upstate Biotechnology) at 4°C overnight, respectively. The immunocomplex was precipitated by protein A/G plus agarose beads. After being washed, the immunoprecipitates were eluted with elution buffer. The eluted immunocomplex was treated with RNase A overnight at 65°C, and the contained proteins were removed by treatment with EDTA, 1 M Tris Cl (pH 6.5), and proteinase K at 42°C for 1 hour. The remaining DNA was extracted using a DNA purification kit (QIAGEN, Germany) and eluted in 50 μL Elution Reagent C. The gene promoter sequences in the immunoprecipitates were amplified by PCR using the primers in Additional File 1.

### Quantitative real-time PCR

RNA was isolated from harvested cells with Trizol reagent (Invitrogen) and then treated with DNase (Roche) to eliminate contaminated DNA, followed by reversely transcribing into cDNA, according to the instructions of Promega. The relative levels of target mRNA transcripts to control β-actin were determined by quantitatively PCR using SYBR green pre-mixture and specific primers (Additional file 1, Supplementary Table 1) in a Bio-Rad IQ5 Real-Time PCR System. The relative levels of miR-185 to control U6 snRNA were also determined by quantitative RT-PCR using the primers in Additional File 1.

**Table 1:** Function and pathway analysis of the promoter hypermethylated genes identified by MeDIP-chip

Term	Promoter Hypermethylation Genes
**Go Term**	
cell communication, intracellular signaling cascade, signal transduction	23 genes: SST, DIRAS3, PTAFR, KCNN3, OR10Q1, CD81, ABRA, CASP9, FYN, MBP, OR10H5, RCVRN, GPR31, KCNMB2, TDGF1, ANKDD1A, OPN1MW, PKP1, PCDHB13, PI4KA, HSH2D,KNDC1, KCNMB3
neurological system process, system process, synaptic transmission, transmission of nerve impulse	21 genes: KCNMB3, CLN3, MPZ, S100P, TRPV1, DLGAP2, SIX3, PI4KA, RCVRN, PCDHB13, OR10H5, MBP, KCNMB2, PROM1, FYN, SYPL1, KCNN3, OR10Q1, HTR1D, SST, GAD1
negative regulation of biological process, negative regulation of metabolic process, negative regulation of cellular process, negative regulation of transcription, transcription repressor activity	16 genes: SST, ST18, B4GALNT2, DEDD2, GRLF1, SALL4, RASSF2, PAIP2, TDGF1, RASSF1, CLN3, GDNF, SIX3, DKK4, TMSB4Y, CFTR
homeostatic process, chemical homeostasis, ion homeostasis, regulation of pH, regulation of biological quality	13 genes: KCNMB3, CCKAR, CLN3, MPZ, TRPV1, CYP11B2, RPH3AL, KCNMB2, MBP, EDNRB, DNAJC16, DEDD2, MB
brain development, generation of neurons, neuron migration	8 genes: FYN, NNAT, CCKAR, ROBO2, SIX3, CFTR, GRLF1, GDNF
cell adhesion, biological adhesion, homophilic cell adhesion, Cadherin	14 genes: FGF6, FLRT1, CLDN18, PKP1, CD300A, FERMT3, SDK1, FBLN7,PCDHB13, ROBO2, PCDHA12, PARVB, PCDHA8, PCDHA13
ion transport, Calcium channel, cation channel activity, gated channel activity, ion transmembrane transporter activity, transmembrane transporter activity	10 genes: KCNMB3, TRPV1, FYN, CACNG7, KCNN3, TRPV3, CACNG1, SLC5A11, KCNK10, KCNMB2
cell migration, cell motility, localization of cell, cellular morphogenesis during differentiation, cellular structure morphogenesis	9 genes: SST, GDNF, ROBO2, S100P, FYN, CCKAR, TDGF1, EDNRB, CFTR
cytoskeletal protein binding, actin binding	7 genes: PARVB, ABRA, C14orf49, RPH3AL, TMSB4Y, PDE4DIP, FYN, PHACTR3
induction of apoptosis by extracellular signals	2 genes: SST, DEDD2
**KEGG_PATHWAY**	
Neuroactive ligand-receptor interaction	7 genes: TRPV1, EDNRB, SST, PTAFR, GH2, CCKAR, HTR1D
MAPK signaling pathway	4 genes: CACNG7, MAP2K3, CACNG1, FGF6
Wnt signaling pathway	1 genes: DKK4
Jak-STAT signaling pathway	1 genes: GH2

### Western blotting

Western blot was performed as previously described [35]. Protein extracts (30 μg) were resolved on 10% SDS-polyacrylamide gels. The proteins were transferred onto PVDF membranes, incubated with 5% skim milk at room temperature in TTBS (20 mM Tris-HCl, pH 7.5, 500 mM NaCl, 0.1% Tween-20), and then incubated at 4°C for 12 h with rabbit polyclonal primary antibody against DNMT1 and mouse monoclonal antibody GAPDH from Cell Signaling Technology (Beverly, MA, USA). DNMT1 and GAPDH antibodies were diluted 1:1000, respectively. After washed with TTBS, the membranes were incubated at 37°C for 1 h with goat anti-rabbit IgG and goat anti-mouse IgG secondary antibodies diluted 1:1000 (Boster Biological Technology). The membranes were developed using the chemiluminescent substrate ECL detection system (Amersham) and bands were visualized on X-ray film (Kodak).

### Luciferase assays

The 53 bp of 3'UTR of the DNMT1 gene were synthesized and annealed, then inserted into the *Hind III *(aagctt) and *Spe I *(actagt) sites of pMIR-REPORT(TM) Luciferase vector (Ambion) at downstream of the stop codon of the gene for luciferase. The sequences were: sense 5'-ctagtTTTATGTAGTTTTTATATGTTGTAATATTTCTTCAAATAAATCTCTCCTATAAa-3'and anti-sense 5'-agcttTTATAGGAGAGATTTATTTGAAGAAATATTACAACATATAAAAACTACATAAAa-3' for DNMT1. We also generated a mutant with 4 bp deletion (TCTC) at the potential binding sequence of miR-185 of the 3'UTR of the DNMT1 gene. U251 cells were co-transfected with 0.8 μg the generated firefly luciferase report vector and 0.2 μg the Psv-β-galactosidase control vector (Promega) using Lipofectamine 2000 (Invitrogen) according to the manufacturer's protocol. Firefly luciferase activities were measured using the Luciferase Assays System (Promega) 48 hours after transfection. β-galactosidase activity was measured using β-galactosidase Enzyme Assay System (Promega).

### DNMT1 silencing by siRNA

A siRNA oligonucleotide (5'-UUUGAUGUCAGUCUCAUUGGG-3') targeting *DNMT1 *was designed as siDNMT1 and synthesized by Invitrogen. Scrambled siRNA was used as a negative control. U251, SF126, and SF767 glioma cells were cultured in dishes or 96-well plates for 24 h, and transfected with siDNMT1 or control scrambled siRNA, using Lipofectamine 2000 (Invitrogen) for 48 h. The cells were then subjected to further assays or to RNA and protein extraction.

### GDM analysis

Genomic DNA was isolated from U251, SF126, and SF767 glioma cells using a genomic DNA extraction kit, according to the manufacturer's instructions (TaKaRa). The contents of global DNA methylation in individual samples were determined by high performance liquid chromatography/diode array detectors (HPLC-DAD), as previously described [36]. Briefly, 1 mg genomic DNA was first denatured at 100°C for 3 min and the denatured DNA was treated with nuclease P1 in the presence of 0.01 M ammonium acetate (pH 5.3) at 45°C for 2 h. Subsequently, the DNA samples were treated with venom phosphodiesterase I in the presence of 0.1 M ammonium bicarbonate at 37°C for 1 h and with alkaline phosphatase at 37°C for 1 h. The DNA samples were characterized by a Phenomenex C18 column (250 mm × 4.6 mm, 5 μm) with ammonium formate-methanol (3:7) as mobile phase at the flow rate of 1 mL/min, the column temperature at 20°C, and UV detection wavelength at 260 nm.

### Microsatellite markers and LOH analysis of the miR-185 locus

The five polymorphic microsatellite markers on the chromosome 22q11, including the has-miR-185, were screened in 12 glioma specimens and their blood samples by PCR using fluorescent dye-labeled forward primer and unlabeled reverse primer (invitrogen). The investigated markers and their chromosomal locations are shown in Additional File 2. PCR amplifications were performed in 10 μL of reaction volumes containing 50 ng of genomic DNA, 5 μL of PCR Premix Tag (TaKaRa), 4 pmol of fluorescent dye-labeled forward primer, and 4 pmol of unlabeled reverse primer, according to the manufacturers' instruction: denaturation at 95°C for 10 min; 18 cycles at 94°C for 30 s, 63-54°C for 30 s (Temperature decrease 0.5°C per cycle), and 72°C for 1 min; 18 cycles at 94°C for 30 s, 54°C for 30 s, and 72°C for 1 min; and a final extension at 72°C for 10 min. Each 10 μL sample of the resulting PCR products was diluted with 20 μL of H2O, and a 1.0 μL aliquot of each diluted fluorescent-labeled PCR product was combined with 12 μL of formamide and 0.5 μL of GeneScan™400 HD standard (Applied Biosystems). Capillary electrophoresis was then performed using an ABI 310 DNA Analyzer, and the results were analyzed using GeneMapper software (Applied Biosystems). Allelic loss at each microsatellite locus was considered to be present in tumor samples' DNA when there was at least a 65% peak reduction at one of a pair peak compared with the corresponding peak of normal DNA.

### Statistical analysis

The levels of methylation in the specific gene of individual samples were calculated, according to the percentage of methylation in all CpG sites of the target promoter regions. The difference in the levels of methylation between glioma samples and non-tumor subjects was analyzed by One-way ANOVA, and the difference in the levels of gene expression and miR-185 in different cell lines and tissue samples was analyzed by Student's *t*-test using SPSS 10.0. The potential correlation between gene expression and methylation levels was determined by Pearson correlation and linear regression analysis. A p value < 0.05 was considered statistically significant.

## Results

### Identification of differential methylation regions in whole genome between primary glioma and non-tumor brain tissues

To better understand the global changes in the levels of DNA methylation in primary glioma, six primary gliomas (one astrocytoma grade I, three astrocytoma samples at grade II, and two astrocytoma samples at grade III) from both male and female patients and four age- and gender-matched non-tumor brain samples were subjected to analysis of genome-wide methylation. The methylated DNA fragments in the genome of each sample were enriched by MeDIP and the whole-genome interrogations were hybridized to the HG18 CpG promoter microarrays that cover 28,226 CpG islands and 17,000 reference gene promoter regions of the entire human genome.

**Figure 1:**
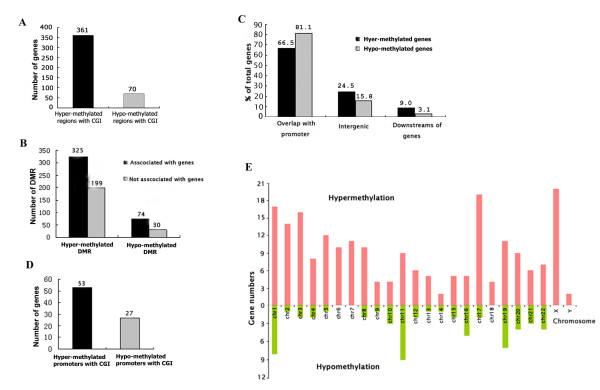
**Genome-wide analysis of the differentially methylated regions (DMRs) in primary glioma**. (A) The numbers of DMRs associated with CpG islands (CGI). (B) The number of DMRs associated with, or without, genes. (C) The distribution of DMRs associated with genes. Most of the identified DMRs associated with genes were mapped to gene promoters. (D) The number of DMRs in the both gene promoters and CpG islands. (E) The chromosomal distribution of 216 promoter hypermethylated genes and 60 promoter hypomethylated genes.

524 hypermethylated and 104 hypomethylated regions were indentified in the primary gliomas. Among these regions, 361 hypermethylated and 70 hypomethylated regions were CpG islands (Figure 1A). Intriguingly, 325 hypermethylation and 74 hypomethylation regions were mapped to annotated gene regions, including the promoter, intragenic, and downstream of genes. However, 199 hypermethylated and 30 hypomethylated regions were mapped to the genomic regions without any gene annotation (Figure 1B). Notably, 216 out of 325 (66.5%) of hypermethylated and 60 out of 74 (81.10%) of hypomethylated regions were mapped to the promoter regions of known genes (Figure 1C). Some hypermethylated (53) and hypomethylated (27) regions were mapped to both the promoters of known genes and CpG islands (Figure 1D). Thus, there are many novel differential methylation regions (DMRs) in the promoters, intragenic, downstream of known genes, and unannotated genomic regions in primary gliomas.

The methylation status in the promoter is associated with the regulation of gene expression and tumor development. We further analyzed the DMRs mapped to the gene promoters in gliomas, and the 216 hypermethylated and 60 hypomethylated promoters identified by MeDIP-chip were distributed in different loci and chromosomes (Figure 1E, Additional File 3, and Additional File 4). While the promoter-hypermethylated genes were present predominately in the 1, 2, 3, 17, and x chromosomes, small numbers of the promoter-hypermethylated genes occurred in other chromosomes. Interestingly, the promoter-hypomethylated genes were mainly distributed in the 1, 11, 16, 19, 20, and 22 chromosomes.

We also performed functional and pathway analysis of these differential promoter- methylated genes using the DAVID bioinformatics tools (Table 1 and 2) [37]. The majority of the differential promoter-methylated genes were clustered into several networks, and involved in a wide variety of biological functions, including cell communication, the neurological process, negative regulation of the biological process, the homeostatic process, brain development, cell adhesion, ion transport, cytoskeletal protein binding, regulation of transcription, and apoptosis. Some of the genes, such as events in the MAPK, Wnt, and Jak-STAT signal pathways, were also involved in regulating the development of tumors. In addition, several of the promoter-hypermethylated genes were associated with the development of human cancers and these include GIPC2, DIRAS3, TYSPL6, EDNRB, FYN, GDNF, RASSF1, and RASSF2 [38-43]. Apparently, the research strategies and resulting data are valuable for the evaluation of the significance of DMRs in gliomas.

**Table 2:** Function and pathway analysis of the promoter hypomethylated genes identified by MeDIP-chip

Term	Promoter Hypomethylation Genes
**Go Term**	
signal transduction, cell communication	14 genes:OR10G4, BAD, C9, OR51S1, CCRL2, OR8A1, SFN, ABR, OR1L6, FKBP8, DRD4, SORBS1, GP1BB, MLNR
protein metabolic process, cellular metabolic process, biopolymer metabolic process	11 genes: CPEB1, C9, NRBP2, PSMF1, OR51S1, KLHL21, FKBP8, TUBB4Q, PRSS33, FUT5, TUBB8
transport, ion channel activity, metal ion transport, cation transport	10 genes:ACCN1, ABCC12, MFSD3, SLC28A1, SLC5A9, SLC2A9, KCNK4, TUBB4Q, TUBB8, SORBS1
hydrolase activity, serine hydrolase activity	5 genes:ABCC12, OR51S1, TUBB4Q, PRSS33, TUBB8
regulation of gene expression,regulation of transcription,DNA binding,transcription factor activity	5 genes: CPEB1,TOX2, LMO3, PHF13, NAT14
nervous system development, system development, organ development	3 genes: IGSF8, ACCN1, ABR
apoptotic program, induction of apoptosis, cell death	3 genes: BAD, SFN, C9
**KEGG_PATHWAY**	
Neuroactive ligand-receptor interaction	3 genes: SCT, MLNR, DRD4
Insulin signaling pathway	2 genes: BAD, SORBS1

### Validation of the methylation status in the gene promoter of human gliomas

**Figure 2:**
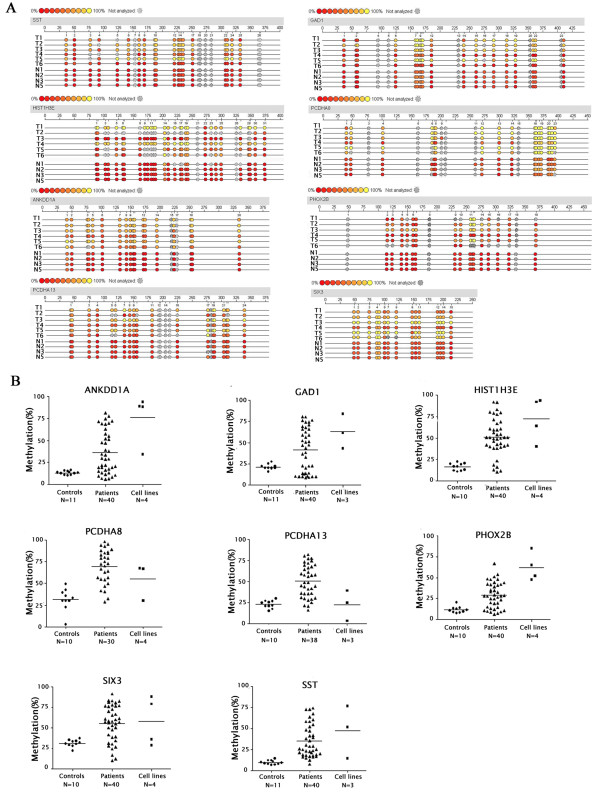
**MassARRAY analysis of methylation in primary gliomas**. (A) The levels of promoter methylation in the hypermethylated genes identified by MeDIP-chip. MassARRAY assay were performed using gDNA from samples screened by microarray. Different colors of circles mark the position of CpG dinucleotides within the sequence (straight line) and the levels of methylation. Gray circles represent the unanalyzed CpG sites. The ruler on the top of each gene sequence indicates the base pair position within the sequence on the top and the positions of the CpG sites at the bottom. For all tested genes, significant hypermethylation in the promoter regions were observed in glioma samples compared with non-tumor controls. T1, T2, T3, T4, T5, and T6 were glioma samples; N1, N2, N3, and N5 were age- and gender-matched non-tumor brain samples. Glioma samples and non-tumor control samples were simultaneously analyzed. (B) The methylation levels of 8 selected genes identified by MeDIP-chip in non-tumor controls, primary glioma patients and glioma cell lines. DNA methylation was analyzed by MassARRAY assay. The name of each gene is shown in the upper part of each panel. N represents the number of cases studied for each gene and each group. Each panel represents the results of an individual gene indicated in the upper of each graph.

To confirm the results of microarray experiments, eight candidates of the promoter-hypermethylated genes, which were never reported in glioma, were selected (Additional File 5) and characterized by the Sequenom's MassARRAY system. Significantly higher levels of hypermethylation in the promoter regions of the genes were observed in glioma samples, as compared with that in normal brain tissue controls (Figure 2A). The degrees of methylation as determined by MassARRAY were correlated well with the results obtained by MeDIP-chip.

To further determine whether these eight gene promoters were hypermethylated in other primary glioma samples and cell lines, we subsequently characterized the methylation status of these eight gene promoters in 4 glioma cell lines, 40 samples from patients with primary glioma, and 11 non-tumor brain samples by MassARRAY (Figure 2B). The methylation levels of seven gene promoters (ANKDD1A, GAD1, SIX3, SST, PHOX2B, PCDHA8, and HIST1H3E) in glioma patients and cell lines were significantly higher than those in non-tumor controls (p < 0.01). The methylation levels in the PCDHA13 promoter in primary glioma samples, but not in glioma cell lines, were also significantly higher than that in non-tumor controls (p < 0.01). A similar pattern of the promoter hypermethylation was achieved by bisulfite sequencing (data not shown). These data suggest that the hypermethylation in the ANKDD1A, GAD1, SIX3, SST, PHOX2B, PCDHA8, HIST1H3E, and PCDHA13 gene promoters may be associated with the development of human glioma. More importantly, the methylation levels of the ANKDD1A and PHOX2B promoters tended to be negatively correlated with age and the differentiation grades of human glioma (Table 3). The promoters of these two genes may be likely methylated in younger patients with low grade of gliomas.

**Table 3:** The methylation of the gene promoters in glioma patients and their clinical characteristics

Variable	Methylation n (%)							
	
	ANKDD1A	GAD1	HIST1H3E	PCDHA8	PCDHA13	PHOX2B	SIX3	SST
**Sex**								
Male	18 (64.3)	15 (53.6)	25 (89.3)	13 (65.0)	20 (76.9)	19 (67.9)	19 (67.9)	27 (96.4)
Female	11 (91.7)	9 (75.0)	9 (75.0)	7 (70.0)	9 (75.0)	10 (83.3)	10 (83.3)	11 (91.7)
***p-*vablue**	0.076	0.205	0.246	0.784	0.897	0.315	0.315	0.527

**Age**								
< 45 y	18 (90.0)	13 (65.0)	17 (85.0)	12 (60.0)	17 (85.0)	18 (90.0)	14 (70.0)	19 (95.00
≥45 y	11 (55.0)	11 (55.0)	17 (85.0)	8 (40.0)	12 (60.0)	11(55.0)	15 (75.0)	19 (95.0)
***p-*vablue**	0.013 *	0.519	1.000	0.121	0.077	0.013 *	0.723	1.000

**Differentiation**								
Grade I-II	20 (90.9)	16 (72.7)	19 (86.4)	14 (63.6)	18 (81.8)	19 (86.4)	18 (81.8)	22 (100.0)
Grade III-IV	9 (50.0)	8 (44.4)	15 (83.3)	6 (54.5)	11 (68.8)	10 (55.6)	11 (68.8)	16 (88.9)
***p-*vablue**	0.004 **	0.069	0.789	0.425	0.350	0.030 *	0.145	0.109

**Tumor type**								
A	22 (73.3)	18 (60.0)	27 (90.0)	15 (68.2)	22 (73.3)	23 (76.7)	20 (66.7)	29 (96.7)
GBM	3 (50.0)	3 (50.0)	4 (66.7)	2 (50.0)	4 (100.0)	3 (50.0)	5 (83.3)	5 (83.3)
OA	4 (100.0)	3 (75.0)	3 (75.0)	3 (75.0)	3 (75.0)	3 (75.0)	4 (100.0)	4 (100.0)
***p-*vablue**	0.217	0.732	0.289	0.723	0.498	0.407	0.304	0.349

*p*-values from Chi-square test, *p-value < 0.05, ** *p*-value < 0.005.

A, Astrocytoma; GBM, Glioblastoma multiforme; OA, Oligoastrocytoma

### Epigenetic regulation of the expression of the promoter-hypermethylated genes

**Figure 3:**
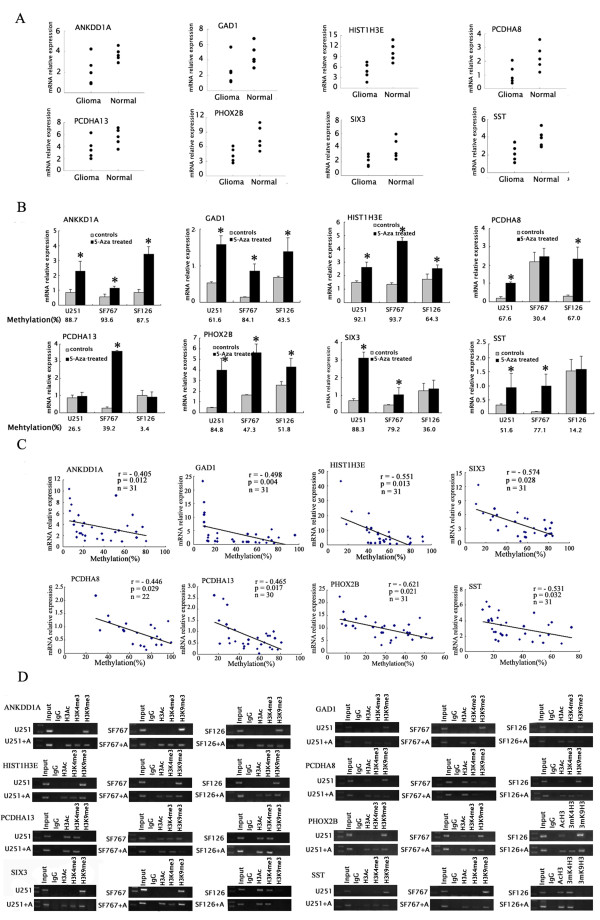
**The expression of the promoter-hypermethylated genes was regulated epigenetically in glioma**. (A) The expression of the promoter-hypermethylated genes in primary gliomas (n = 5) and non-tumor brain tissue samples (n = 5) was detected by quantitative RT-PCR. Data shown are mean value of individuals for each gene from three independent experiments. (B) The expression of the promoter hypermethylated genes in the 5-aza-2'-deoxycytidine-treated glioma cells. Data are expressed as mean% ± SEM of each group of cells for the indicated gene from three separate experiments. *p< 0.05 vs. control. (C) Correlation analysis of the levels of gene promoter methylation with its mRNA expression. The expression was analyzed with real time PCR, while the contents of promoter methylation in primary glioma samples were determined massarrays (n = 22-31 per group). Data shown are mean value of individual samples. (D) CHIP-PCR analysis for histone marks H3Ac (histone H3 acetylation), H3K4me3 (trimethyl-histone H3 lys4), and H3K9me3(trimethyl-histone H3 lys9) in the promoter-hypermethylated genes. U251, SF767, and SF126 cells were untreated or treated with 5-aza-2'-deoxycytidine. Input represents amplification from 1% chromatin before immunoprecipitation. IgG represents negative controls immunoprecipitated by normal rabbit serum. H3Ac, immunoprecipitated by rabbit anti-acytyl-H3; H3K4me3, immunoprecipitated by rabbit anti-trimethyl-H3(lys4); H3K9me3, immunoprecipitated by rabbit anti-trimethyl-H3(lys9).

To examine the role of the promoter methylation in the regulation of gene expression, the relative levels of mRNA transcripts of these eight promoter-hypermethylated genes in several primary glioma and non-tumor brain samples were determined by quantitative RT-PCR. The relative levels of these gene mRNA transcripts were slightly lower in primary glioma tissues than that in non-tumor tissues although it was not significant (Figure 3A). Furthermore, treatment with demethylating agent of 5-aza-2'-deoxycytidine significantly up-regulated the expression of those promoter-hypermethylated genes in U251, SF767, and SF126 cells (p < 0.05). However, treatment with 5-aza-2'-deoxycytidine failed to modulate the expression of SST and SIX3 that were not the promoter-hypermethylated genes in SF126, the PCDHA13 in U251 and SF126, and the PCDHA8 in SF767 cells (Figure 3B). In addition, the expression levels of those promoter-hypermethylated genes were inversely associated with the degrees of their promoter methylation in primary glioma samples (Figure 3C). These data indicate that hypermethylation in the gene promoters down-regulates the expression of these genes, which may contributes to the development of glioma in Chinese patients.

Because there are cross-talks between DNA methylation and histone modification in the regulation of gene expression, we characterized the binding of different histone markers, H3Ac (histone H3 acetylation), H3K4me3 (trimethyl-histone H3 lys4), and H3K9me3 (trimethyl-histone H3 lys9) around the promoter of those genes in U251, SF767, and SF126 cell lines by CHIP. We found that high levels of H3K9me3, but not H3Ac and H3K4me3, bound to the promoter regions of these genes when their promoters were hypermethylated in glioma cells. In contrast, H3Ac and H3K4me3, not H3K9me3 bound to their promoters when they were not methylated in glioma cells. Interestingly, treatment with 5-aza-2'-deoxycytidine decreased H3K9me3, but increased H3Ac and H3K4me3 binding to these gene promoter regions (Figure 3D). Apparently, hypermethylation in the promoters of these genes is associated with the modulation of histone and chromatin structure, and regulation of gene expression in glioma cells.

### Loss of heterozygosity at the miR-185 locus located in the 22q11.2 in glioma

**Figure 4:**
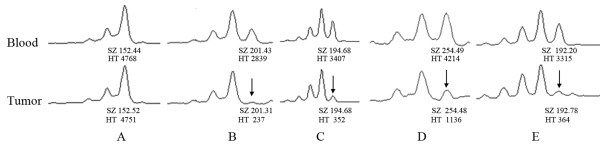
**Microsatellite analysis of the miR-185 locus in 22q11.2 in glioma and paired blood samples**. (A) LOH analysis at D22S420, 22q11.1. (B) LOH at D22S446, 22q11.21. (C) LOH at D22S686, 22q11.22. (D) LOH at D22S925, 22q11.23. (E) LOH at D22S315, 22q12.1. Each peak has a box that provides the fragment size and peak height (upper and lower labels, respectively). The arrow indicates the allele loss.

Previously studies have reported that microRNA-185 expression is down-regulated in glioma [44,45]. However, the mechanism underlying down-regulated miRNA-185 expression in glioma is uncovered. MiR-185 is located in the 22q11 chromosome region and loss of heterozygosity (LOH) is observed in several types of cancers [46,47]. Whether the miR-185 locus in the 22q11.2 in glioma could be subjected to the LOH in glioma is still unknown. Therefore, we performed LOH analysis using 5 microsatellite markers spanning the 22q11 (Additional file 2, Supplementary Table 2). The partial results of LOH analysis spanning the 22q11 are shown in Figure 4. We observed five out of 12 cases with LOH in the 22q11.21 (D22S446), four in the 22q11.22 (D22S686), and six in the 22q11.23 (D22S925), but no single one in the 22q11.1 region (D22S420), respectively. In addition, we detected three cases with LOH in the 22q12.1 (D22S315).

### DNTM1 is the target of miR-185

**Figure 5:**
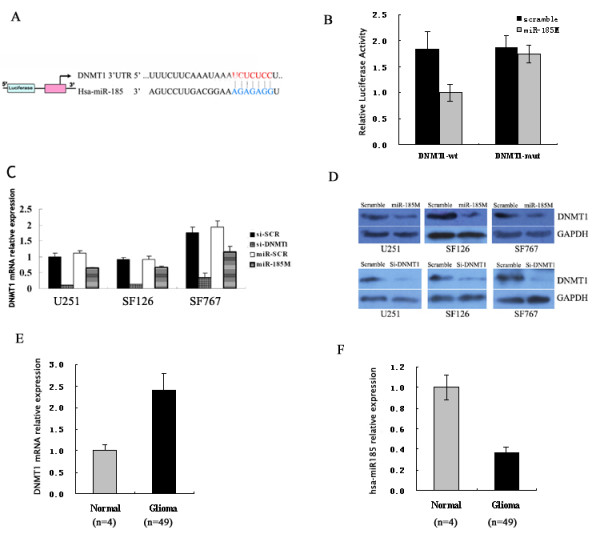
**MicroRNA-185 targets DNMT1 mRNA at the 3'-UTR**. (A) Schema of the firefly luciferase reporter constructs for the DNMT1, indicating the interaction sites between miR-185 and the 3'-UTRs of the DNMT1. (B) Luciferase activities. U251 cells were co-transfected with firefly luciferase constructs containing the DNMT1 wild-type or mutated 3-UTRs and miR-185 or scrambled oligonucleotides, respectively. The firefly luciferase activities were measured and normalized to β-galactosidase activity. Data are expressed as mean ± SD of relative levels of luciferase activity of each group of cells from three independent experiments. (C) Quantitative RT-PCR analysis of DNMT1 expression. Glioma cells were transfected with miR-185, si-DNMT1 or controls for 48 h. The levels of DMNT1 mRNA transcripts were determined by Real time PCR. Data are expressed as mean ± SD of each group of cells from three independent experiments. (D) Western blot analysis of DNMT1 protein. The levels of DNMT1 expression in the transfected cells described above were characterized by Western blot assays. Data shown are representative images of each group of cells from three separate experiments. (E) The levels of DNMT1 mRNA transcripts in normal brain tissue and primary gliomas. (F) The levels of miR-185 expression in normal brain tissue and primary gliomas. Data are expressed as mean ± SD of each group of samples from three separate experiments. Then indicates the sample size of each group. *p< 0.05 vs. control.

The DNMT1 has been thought to be a putative target of miR-185 [48] (Figure 5A). To demonstrate this in glioma cells, the DNMT1 complementary sequence or the mutant with a deletion of 4 nucleotides (UCUC) for the predicted binding of miR-185 were cloned downstream of the firefly luciferase gene. U251 cells were co-transfected with the wild or mutated construct, together with miR-185 or scrambled oligonucleotide for 24 h, and luciferase activities in those cells were measured. As shown in Figure 5B, significantly reduced levels of luciferase activities were detected in the cells transfected with the DNMT1 complementary sequences and miR-185, but not in the cells with the mutant sequence and miR-185, indicating that the DNMT1 complementary sequence contained the binding site for miR-185. Furthermore, transfection with miR-185 significantly reduced the levels of DNMT1 mRNA transcripts and protein expression in glioma cells (Figure 5C and 5D), but did not affect the expression of DNMT3A and DNMT3B (data not shown). More importantly, higher levels of DNMT1 mRNA transcripts, but lower levels of miR-185 were detected in primary glioma tissues, as compared with that in non-tumor brain tissues (Figure 5E, F). Collectively, these data indicate that the down-regulated miR-185 expression is related to high levels of DNMT1 expression, which may be associated the development of glioma and support the notion that miR-185 directly targets DMNT1 mRNA, thereby regulating the expression of DNMT1 in glioma cells.

### Over-expression of miR-185 reduces global DNA methylation and induces the expression of the promoter-hypermethylated genes

**Figure 6:**
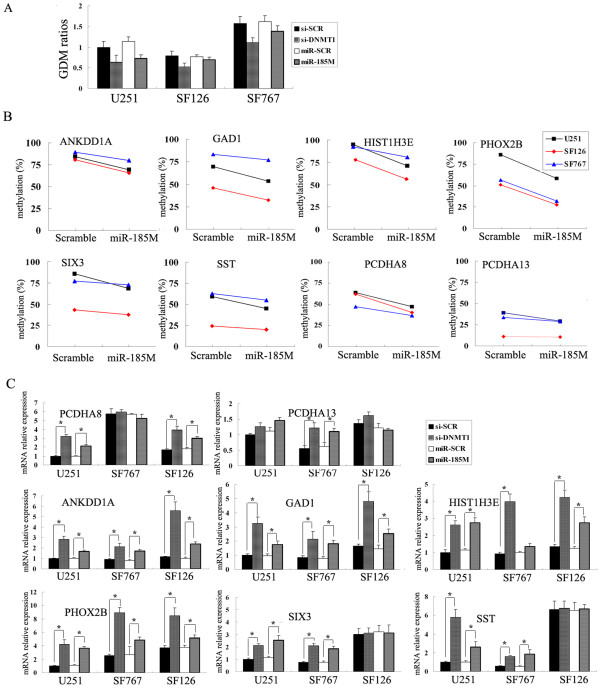
**MicroRNA-185 reduces the levels of GDM and induces the expression of the promoter-hypermethylated genes**. (A) Over-expression of miR-185 reduced the levels of GDM in glioma cell lines. U251, SF126, and SF767 cells were transfected with miR-185 mimics (miR-185M), si-DNMT1, or controls for 48 h and the levels of GDM were determined by HPLC-DAD. Data are expressed as mean ± SD of the relative levels of GDM in each group of cells from three separate experiments. (B) The levels of promoter methylation in the promoter-hypermethylated genes in glioma cell lines. The indicated cells were transfected with miR-185 mimics or control, and the contents of promoter methylation were characterized by MassARRAY system. Data are expressed mean % of the methylated promoters in the indicated genes in those cells. The levels of promoter methylation in these genes were similar to that in unmanipulated cells (data not shown). (C) Quantitative RT-PCR analysis of the relative levels of each gene expression in the indicated cells after transfection with miR-185 mimics, si-DNMT1, or control for 48 h. Data are expressed as mean ± SD of the relative levels of the indicated gene in different cells from three independent experiments. *p< 0.05 vs. control

Given that low levels of miR-185 were associated with higher levels of DNMT1, a key factor for the maintenance of global DNA methylation in mammal cells, we further investigated whether the enforced expression of miR-185 could functionally modulate DNA hypermethylation in glioma cells. U251, SF126, and SF767 cells were transfected with miR-185 mimics, scrambled oligonucleotides, si-DNMT1 sequence, or control siSCR, respectively, and the status of GDM was measured by HPLC-DAD. Transfection with miR-185 mimics, like with siDNMT, reduced the ratio of GDM by 20-30% in U251 cells, as compared with that in the controls (Figure 6A). Further analysis of the promoter-hypermethylated genes revealed that transfection with miR-185 mimics significantly reduced the frequency of methylation in these gene promoters (Figure 6B), accompanied by elevating the relative levels of mRNA transcripts of those genes in the cells (Figure 6C). For example, U251 cells displayed relatively higher methylation in the PCDHA8, ANKDD1A, GAD1, HIST1H3E, PHOX2B, SIX3, and SST genes, and transfection of U251 with miR-185 reduced the methylation levels of these gene promoters and increased the expression levels of these genes. Furthermore, SF126 cells had higher methylation in the PCDHA8, ANKDD1A, GAD1, HIST1H3E and PHOX2B, but lower methylation in the PCDHA13, SIX3, and SST genes, and transfection of the cells with miR-185 only changed the methylation and expression levels of the PCDHA8, ANKDD1A, GAD1, HIST1H3E, and PHOX2B genes, but did not affect the low methylated PCDHA13, SIX3, and SST genes. Apparently, the modulation effects of miR-185 were dependent on the methylation status of individual gene promoters in each cell line.

## Discussion

In this study, we employed the genome-wide CpG promoter microarrays and MeDIP, a sensitive method, to identify the differentially methylated regions in human glioma vs. non-tumor brain tissues. We found numerous differential methylation regions, some of which were validated in a cohort of glioma samples by the Sequenom's MassARRAY system. Hence, the MeDIP-chip platform is both efficient and effective. On the other hand, we had to admit that the data were some limitations because our MeDIP-chip study was based on very small number samples.

We found 524 hyermethylated and 104 hypomethylated regions in glioma. Among them, 216 hypermethylated and 60 hypomethylated regions were mapped to the gene promoters, suggesting that the methylation status may regulate the transcription of these genes. Functional analysis revealed that these promoter-hypermethylated and hypomethylated genes were involved in the regulation of transcription, cell communication, the neurological process, apoptosis, the biological process, the homeostatic process, brain development, cell adhesion, ion transport, and cytoskeletal protein binding. Several of the promoter hypermethylated genes have already been reported in other human cancers, suggesting that they contribute to the tumorigenesis [37-42]. Notably, many of them are novel methylation-regulated genes that have not been indentified previously in human glioma and other malignancies. Apparently, these findings may help in studying the molecular mechanisms underlying the glioma development.

Growing evidence indicates that DNA methylation in the promoter of a gene in cancers seems to be in a nonrandom fashion and the methylation patterns vary, dependent on cell or tumor types, subtypes within the same category of tumors [49]. In addition to the glioma-specific methylation patterns, we identified genomic hotspots, which harbor an abundance of the methylated promoter regions. In particular, we found that the methylation hotspots contained the promoter-hypermethylated genes predominantly on 1, 2, 3, 17, and × chromosomes. A previous study had reported that the methylation hotspots are identified on chromosome 19 in acute lymphoblastic leukemia (ALL) cells [50]. Apparently, the methylation prefers certain regions of genome and these regions are varying in different types of tumors. However, the mechanisms by which various regions of genome are methylated in different types of tumors remain to be further investigated.

We found that the promoters of the ANKDD1A, GAD1, HIST1H3E, PCDHA8, PCDHA13, PHOX2B, SST, and SIX3 genes were hypermethylated in glioma. This is the first report on the promoter hypermethylation in glioma, although the methylation of some of these genes has been reported in other types of caners [51-54]. The ANKDD1A, ankyrin repeat, and death domain-containing protein 1A contain 4 ankyrin repeats that can mediate protein-protein interactions in very diverse families of proteins with apoptosis-related death domains. The ANKRD15 acts as a candidate tumor suppressor in the development of renal cell carcinoma [55]. Given that the hypermethylation of the ANKDD1A promoter occurred predominantly in low grades of glioma, it is possible that the ANKDD1A may also function to be a suppressor in human glioma and inhibit the development of glioma at the early stage. The glutamate decarboxylase 1 (GAD1) encodes glutmatic acid decarboxylase that is responsible for catalyzing L-glutamic acid into gamma-aminobutyric acid, an inhibitory neurotransmitter. The promoter methylation of the GAD1 can inhibit the expression of GAD1 in schizophrenia [56]. HIST1H3E, a member of the histone H3 family, can interact with linker DNA between nucleosomes and functions in the compaction of chromatin into higher order of structures. This protein is differentially expressed in the temporal lobe of patients with schizophrenia [57]. PCDHA8 and PCDHA13 are members of the protocadherin alpha family. These neural cadherin-like cell adhesion proteins are integral plasma membrane proteins that can play a critical role in the establishment and function of specific cell-cell connections in the brain. The protocadherin-gamma subfamily A11 (PCDH-gamma-A11) gene is hypermethylated in astrocytomas [18]. The inactivation of this cell-cell contact molecule may promote the invasiveness of astrocytoma cells. The PHOX2B (paired-like homeobox 2b), a member of the paired family of homeobox, encodes the DNA-associated protein in the nucleus, which functions as a transcription factor involved in the development of major noradrenergic neurons and the determination of neurotransmitter phenotype. PHOX2B can also enhance the second messenger-mediated activation of the dopamine beta-hydrolase, c-fos promoters, and several enhancers, including cyclic AMP-response element and serum-response element. Aberrant methylation of the PHOX2B promoter region accounts for 12.9% of human neuroblastoma cell lines [51]. SST (somatostatin) is expressed throughout the body and can inhibit the release of numerous secondary hormones by binding to the high-affinity G-protein-coupled somatostatin receptors. Somatostatin also regulates the neurotransmission rates in the central nervous system and proliferation of both normal and tumorigenic cells. Recently, SST promoter methylation has been found in various cancers, including gastric cancer, cervical cancer and colon cancer [52,53,58]. SIX3 (SIX homeobox 3) is a member of the sine oculis homeobox transcription factor family. SIX3 regulates the transcriptional activity of the orphan nuclear receptor NOR-1 (NR4A3), which regulates the survival of cells, and acts as a part of the EWS/NOR-1 fusion protein, implicating the oncogenesis of human extraskeletal myxoid chondrosarcoma (EMC) [59]. Together, these novel genes with the hypermethylated promoters in glioma may contribute to the initiation and progression of glioma and potentially serve as a biomarker for the prognosis of human glioma.

In addition, we found that the percentages of promoter methylation in the ANKDD1A and PHOX2B, but not the GAD1, HIST1H3E, PCDHA8, PCDHA13, SIX3 and SST, were associated negatively with the differentiation degrees of human gliomas. These data suggest that ANKDD1A and PHOX2B may play an important role in regulating the early development of glioma and serve as a biomarker for early diagnosis. The GAD1, HIST1H3E, PCDHA8, PCDHA13, SIX3 and SST may regulate the progression of glioma.

DNA methylation in the promoters has been implicated in the down-regulation of gene expression, possibly through the formation of an altered chromatin structure that is resistant to transcription initiation [5,60]. Our study found that the expression of these eight promoter-hypermethylated genes was significantly down-regulated in glioma samples, as compared with that in non-tumor brain samples. More importantly, the contents of promoter methylation in these genes were inversely correlated with the expression levels of these genes in glioma. Furthermore, treatment with 5-aza-2'-deoxycytidine re-activated the expression of promoter-hyermethylated genes in glioma cells *in vitro*. Notably, the DNA methylation is associated with the histone modification, contributing to the gene regulation and to the establishment and maintenance of chromosomal domains. Indeed, we found that the H3K9me3 was enriched, while the H3 acetylation and H3K4me3 were down-regulated in the promoter-hypermethylated genes of glioma cells. Treatment with 5-aza-2'-deoxycytidine reduced the levels of H3K9me3, but increased the H3 acetylation and H3K4me3 in the promoter region of these genes. These data suggest that the H3K9me3 is directly associated with the promoter methylation, while the H3 acetylation and the 3mK4H3 is inversely related to the promoter methylation in glioma cells.

Deletion of genomic interval encompassing miR-185 (22q11.2) is an extremely frequent event in diverse types of cancers [46,47]. The miR-185 can induce cell cycle arrest in human non-small cell lung cancer [61]. In this study, we found that the expression of miR-185 was significantly down-regulated in glioma, as compared with that in non-tumor brain tissues. Moreover, the LOH status was found at the miR-185 locus located in the 22q11.2. Thus, the reduced levels of miR-185 expression may be associated with the loss of 22q11.2 in glioma. These data support that the loss of miR-185 is also a frequent event in glioma and suggest that loss of miR-185 may contribute to the development of human glioma.

Furthermore, we also characterized the role of miR-185 in the regulation of DNA methylation in glioma. We found that transfection with miR-185 and the DNMT1 complementary sequence, but not the mutant with deletion of the miR-185 binding sequence, dramatically reduced luciferase activity in glioma cells, and the levels of DNMT1 expression were inversely correlated with the levels of miR-185 expression in gliomas. In addition, over-expression of miR-185 re-activated the expression of the promoter-hypermethylated genes in glioma cells. These data indicated that miR-185 directly interacted with the DNMT1 and the lower levels of miR-185 expression promoted the abnormal expression of DNMT1 in glioma. Given that DNMT1 is a major player in DNA methylation, higher levels of DNMT1 expression should be responsible for the intensive methylation of these gene promoters, which down-regulate the expression of these genes and contribute to the development and progression of glioma. Therefore, the down-regulated expression of miR-185 and up-regulated expression of DNMT1 contribute to aberrant DNA methylation and in turn to gliomagenesis.

## Conclusions

In summary, we identified aberrant methylation in glioma by MeDIP-chip and found that eight novel genes were epigenetically regulated by DNA methylation in glioma. Our data indicated that miR-185 directly interacted with the DNMT1 and the lower levels of miR-185 expression in glioma may be one of the reasons for the abnormal expression of DNMT1, which leads to aberrant DNA methylation, contributing to the development of human glioma. Apparently, our findings may provide new insights into understanding the pathogenesis of glioma.

## Competing interests

The authors declare that they have no competing interests.

## Authors' contributions

ZPZ and HLT carried out the molecular work, statistical analysis, data interpretation and wrote the initial draft of the manuscript. BXZ and ZYW contributed to patient recruitment, obtained consent, surgically samples collection and handling, gDNA extraction. WL carried out Chromatin immunoprecipitation. HML and LX were involved in GDM analysis, Microsatellite markers and LOH analysis. XPL and RW participated in quantitative real-time PCR and data interpretation. XLL, MHW and GYL supervised the molecular work, participated in data interpretation and revised the manuscript. All authors provided comments of various drafts, participated in direction setting discussions and reviews and have read and approved the final version.

## Supplementary Material

Additional File 1 The sequences of the primers. Click here for file

Additional File 2 Characteristics of polymorphic loci at the 16q22 region. Click here for file

Additional File 3 **Chromosomal localization of 216 promoter hypermethylated genes identified by MeDIP-chip**. The top number indicates chromosome and bottom number indicates the promoter hypermethylated genes identified in each chromosome. The gene names are indicated beside each hypermethylated locus. Click here for file

Additional File 4 **Chromosomal localization of 60 promoter hypomethylated genes identified by MeDIP-chip**. The top number indicates chromosome and bottom number indicates the promoter hypomethylated genes identified in each chromosome. The gene names are indicated beside each hypomethylated locus. Click here for file

Additional File 5 **Examples of 8 promoter hypermethylated genes methylation array profiles**. The green boxes represent normal brain white matter samples (N1, N2, N3, and N5). The red boxes represent the glioma primary samples (T1, T2, T3, T4, T5, and T6). The black bars indicate the regions analyzed by MassARRAY assay. Click here for file
