# Supplementary material for: MiR-185 Targets the DNA Methyltransferases 1 and Regulates Global DNA Methylation in human glioma
Source: Mol Cancer. 2011 Sep 30;10:124. doi: 10.1186/1476-4598-10-124 (PMC3193026; doi:10.1186/1476-4598-10-124)
Supplement: Additional File 1 — The sequences of the primers. [file 1476-4598-10-124-S1.DOC]

**Additional file 1. The sequences of the primers**

|  | **Forward primer(5’-3’)** | **Reverse primer (5’-3’)** | **Annealing Temperature (℃)** | **Product size (bp)** |
| --- | --- | --- | --- | --- |
| **Real time** |  |  |  |  |
| ANKDD1A | CACGTCTGTGTTTTGGGATG | GTGCAGTAAGGTCAGGCCAT | 60 | 131 |
| GAD1 | GCTGTCGCAGAGCCGAGGTG | CCCGCGTTCGAGGAGGTTGC | 60 | 131 |
| HIST1H3E | CCTACTTGGTGGGGCTTTTC | GCTCTGAGAAGAGCCTTTGG | 58 | 161 |
| PCDHA8 | GGGATCAGTTGATGTAGGCG | AATGCCAGCCTCCTCTAGGT | 60 | 142 |
| PCDHA13 | TCCTCCTTGTCTGGGTTCTG | AATGCCAGCCTCCTCTAGGT | 60 | 154 |
| PHOX2B | CGGGAGGAGCTGGCCCTGAA | GCCCGAGGAGCCGTTCTTGG | 58 | 138 |
| SST | CCAACCAGACGGAGAATGAT | CCATAGCCGGGTTTGAGTTA | 58 | 111 |
| SIX3 | AGACGCATTGCTTCAAGGAG | TGCCTACTTGTGTGGGAGTG | 58 | 129 |
| DNMT1 | CTACCAGGGAGAAGGACAGG | CTCACAGACGCCACATCG | 62 | 152 |
| β-actin | AGCGAGCATCCCCCAAAGTT | GGGCACGAAGGCTCATCATT | 62 | 260 |
| miR-185 | CAATGGAGAGAAAGGCAGTTCC | AATCCATGAGAGATCCCTACCG | 62 | 85 |
| U6 snRNA | ATTGGAACGATACAGAGAAGATT | GGAACGCTTCACGAATTTG | 62 | 85 |
| **CHIP** |  |  |  |  |
| ANKDD1A | CAGCCCATCTCAGGTTACACTAC | GCTGTAAATACCGTAATTCCCTCC | 60 | 124 |
| GAD1 | GTTTCCTACGTGGAGCAGACAC | ACTACTGTTTTGTCGCCTTGTCTC | 60 | 160 |
| HIST1H3E | GCGGCGTGAAGAAGCCCCAT | CGCGGAACTCTGGAAGCGCA | 60 | 167 |
| PCDHA8 | GGCGCGTCCGATGCAGATGT | AAGTGGTGCGCAGGAGCGTC | 60 | 164 |
| PCDHA13 | GCCCAAGACCGCGGCAACTA | CAGCCCCAGGTCCTGAGCGA | 60 | 140 |
| PHOX2B | CCTCGGTAGGTCCGGCTCCC | TGCCGCGGACGCAGTAATGG | 60 | 156 |
| SST | CGCCGAGATGCTGTCCTGCC | CGGCAGCAGCCAGGGACTTC | 60 | 134 |
| SIX3 | GGAAGGATAGGACCATCATCTAGG | CCATGACCTCCCCAGACATA | 60 | 167 |
