# Supplementary material for: MiR-185 Targets the DNA Methyltransferases 1 and Regulates Global DNA Methylation in human glioma
Source: Mol Cancer. 2011 Sep 30;10:124. doi: 10.1186/1476-4598-10-124 (PMC3193026; doi:10.1186/1476-4598-10-124)
Supplement: Additional File 2 — Characteristics of polymorphic loci at the 16q22 region. [file 1476-4598-10-124-S2.DOC]

| No | Locus | D number | primers | Product size (bp) | map (bp) | Mark | Genetic Microsatellite status |
| --- | --- | --- | --- | --- | --- | --- | --- |
| 1 | 22q11.1 | D22S420 | F: TGTTCTACACTGAAAATTCTGACGG  R: GAGGGCGTTATCCATGACC | 148-164 | 17859281- 17859644 | AFM217xf4 | Heterozygous |
| 2 | 22q11.21 | D22S446 | F: CCGGAACTTTGGAAGG  R: CCACTTGGGTAACCACTG | 198-232 | 22019111- 22019466 | AFM292va9 | Heterozygous |
| 3 | 22q11.22 | D22S686 | F: TTGATTACAGAGTGGCTCTGG  R: TAAGCCCTGTTAGCACCACT | 180-220 | 23068516-23068798 | GGAA10F06 | Heterozygous |
| 4 | 22q11.23 | D22S925 | F: CTGGCCCCCATCCAAC  R: GCGAGTGATTTCTTATTTCCTGCTT | 247-263 | 25781679- 25781999 | AFMa134yf9 | Heterozygous |
| 5 | 22q12.1 | D22S315 | F: TGCCTATTAAACTCTCCACTCCTTA  R: GCATTATGATTCATTTCTCACAGA | 177-203 | 26015840- 26016079 | AFM183xe9 | Heterozygous |

**Additional file 2. Characteristics of polymorphic loci at the 16q22 region**
