# Supplementary figures and images for: MiR-185 Targets the DNA Methyltransferases 1 and Regulates Global DNA Methylation in human glioma
Source: Mol Cancer. 2011 Sep 30;10:124. doi: 10.1186/1476-4598-10-124 (PMC3193026; doi:10.1186/1476-4598-10-124)

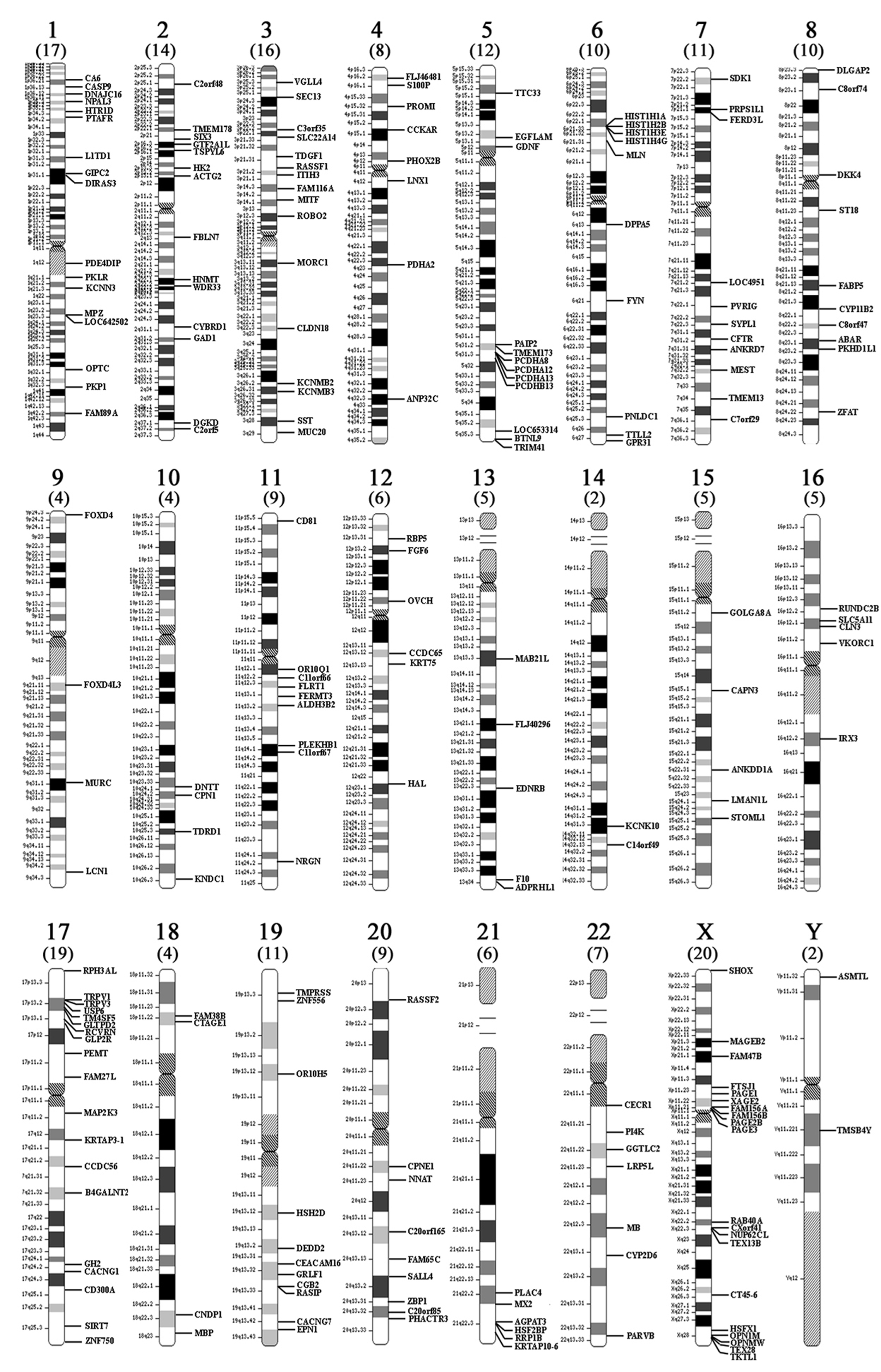

Supplement: Additional File 3 — Chromosomal localization of 216 promoter hypermethylated genes identified by MeDIP-chip. The top number indicates chromosome and bottom number indicates the promoter hypermethylated genes identified in each chromosome. The gene names are indicated beside each hypermethylated locus. [file 1476-4598-10-124-S3.JPEG]

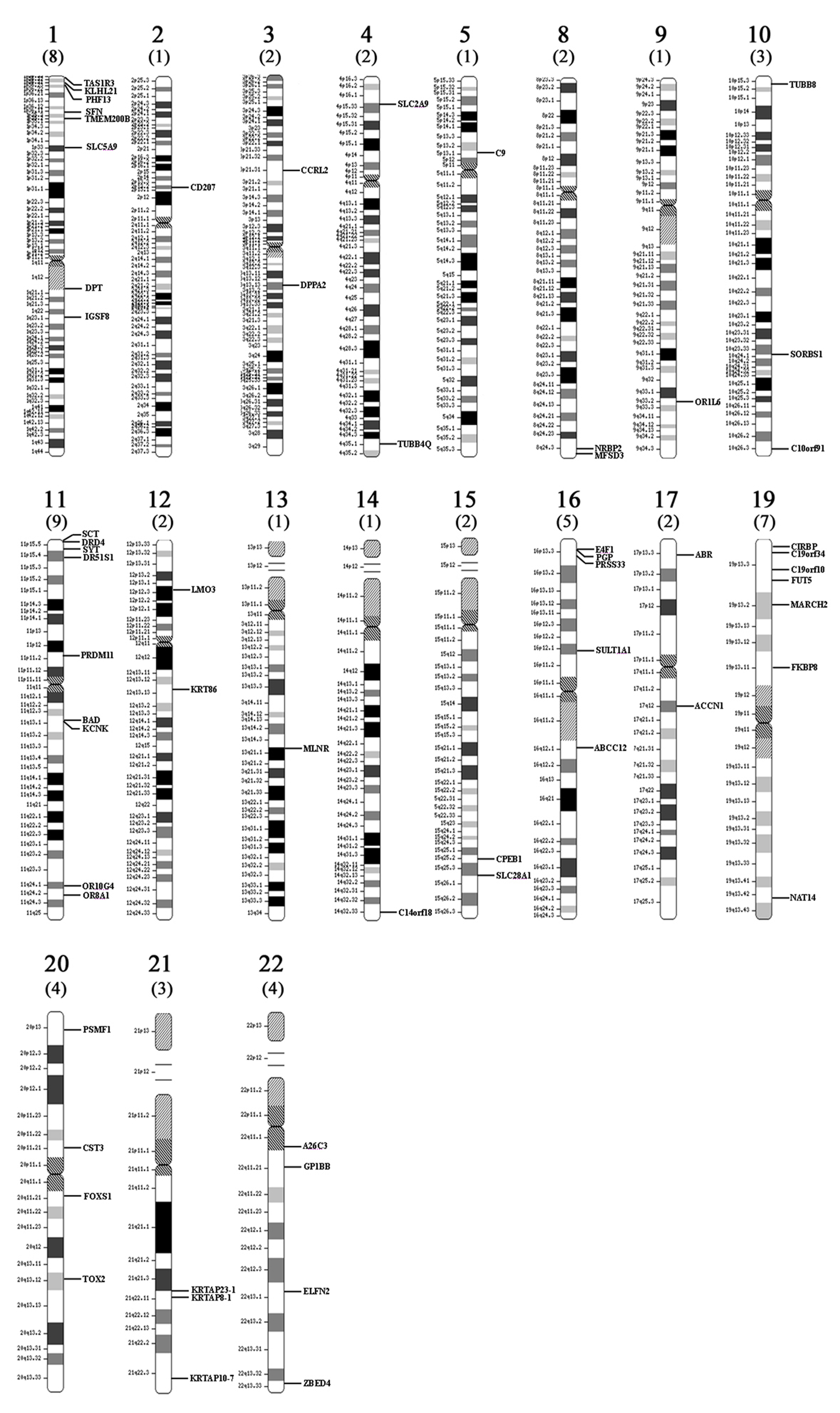

Supplement: Additional File 4 — Chromosomal localization of 60 promoter hypomethylated genes identified by MeDIP-chip. The top number indicates chromosome and bottom number indicates the promoter hypomethylated genes identified in each chromosome. The gene names are indicated beside each hypomethylated locus. [file 1476-4598-10-124-S4.JPEG]

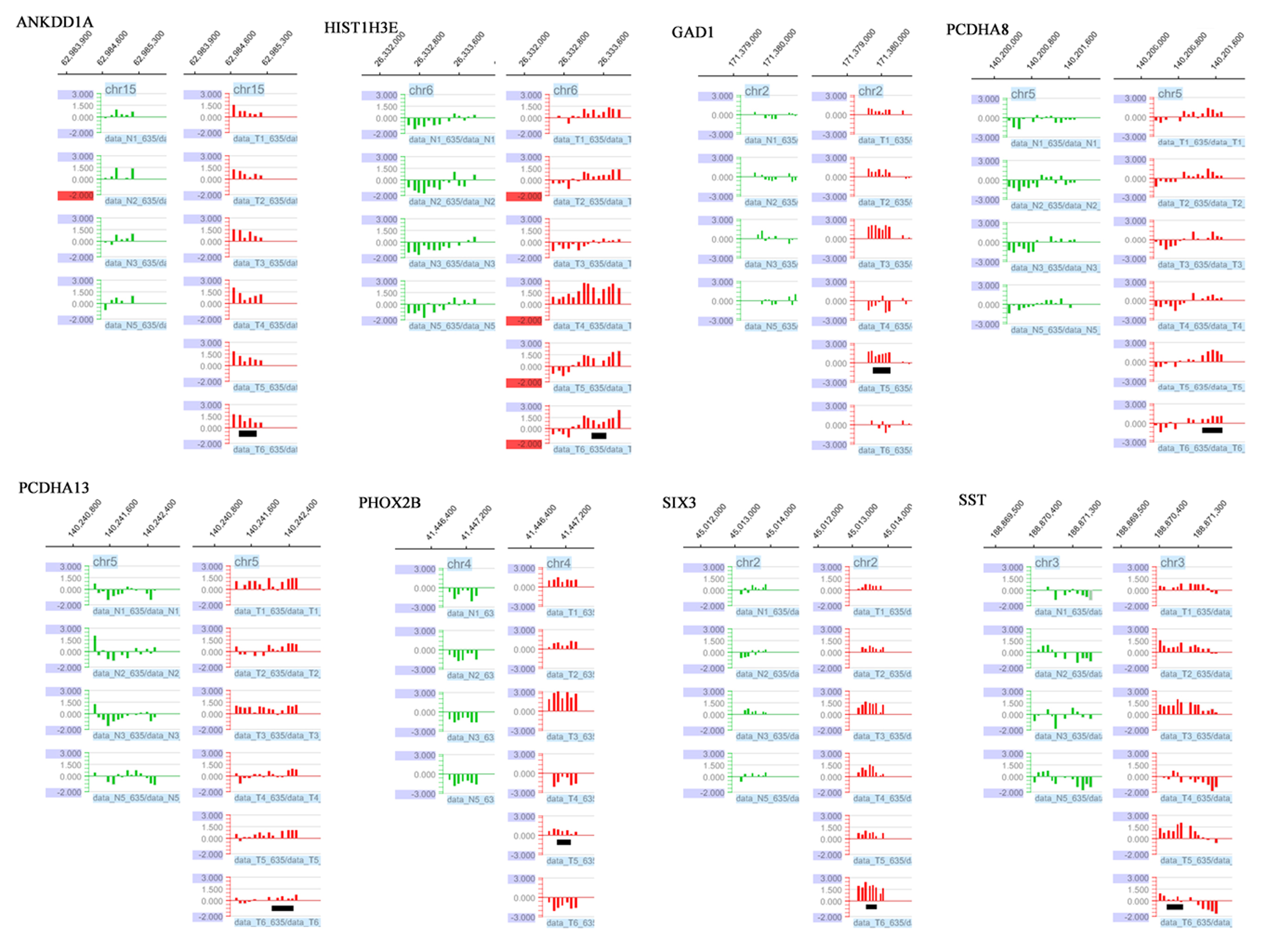

Supplement: Additional File 5 — Examples of 8 promoter hypermethylated genes methylation array profiles. The green boxes represent normal brain white matter samples (N1, N2, N3, and N5). The red boxes represent the glioma primary samples (T1, T2, T3, T4, T5, and T6). The black bars indicate the regions analyzed by MassARRAY assay. [file 1476-4598-10-124-S5.JPEG]
